# The Protective Effects of L-Theanine against Epigallocatechin Gallate-Induced Acute Liver Injury in Mice

**DOI:** 10.3390/foods13071121

**Published:** 2024-04-07

**Authors:** Kun Zhu, Hongzhe Zeng, Lin Yue, Jianan Huang, Jie Ouyang, Zhonghua Liu

**Affiliations:** 1 Xiangya School of Pharmaceutical Sciences, Central South University, Changsha 410013, China; zhukun@csu.edu.cn; 2 Key Laboratory of Tea Science of Ministry of Education, Hunan Agricultural University, Changsha 410128, China; zenghongzhe@hotmail.com (H.Z.); yuelin@saas.ac.cn (L.Y.); jian7513@hunau.edu.cn (J.H.); 3 Hunan Agricultural Product Processing Institute, Hunan Academy of Agricultural Sciences, Changsha 410125, China

**Keywords:** L-theanine, EGCG, liver injury, oxidative stress, metabolomics

## Abstract

Epigallocatechin-3-gallate (EGCG) is a main bioactive constituent in green tea. Being a redox-active polyphenol, high-dose EGCG exhibits pro-oxidative activity and could cause liver injury. L-theanine is a unique non-protein amino acid in green tea and could provide liver-protective effects. The purpose of this study was to investigate the hepatoprotective effects of L-theanine on EGCG-induced liver injury and the underlying mechanisms. A total of 300 mg/kg L-theanine was administrated to ICR mice for 7 days. Then, the acute liver injury model was established through intragastric administration of 1000 mg/kg EGCG. Pretreatment with L-theanine significantly alleviated the oxidative stress and inflammatory response caused by high-dose EGCG through modulation of Nrf2 signaling and glutathione homeostasis. Furthermore, metabolomic results revealed that L-theanine protects mice from EGCG-induced liver injury mainly through the regulation of amino acid metabolism, especially tryptophan metabolism. These findings could provide valuable insights into the potential therapeutic applications of L-theanine and highlight the importance of the interactions between dietary components.

## 1. Introduction

Catechins are the characteristic polyphenols in green tea, usually making up 25–40% of its dry weight [1,2]. The most abundant catechin in tea is epigallocatechin-3-gallate (EGCG), which has been found to perform various biological functions, including antioxidant and anti-inflammatory effects [3]. EGCG is a typical flavan-3-ol phenolic compound with eight free hydroxyl groups in the A ring, B ring, and D ring (Figure S1A), resulting in its redox activity [4]. However, high doses of EGCG have been shown to induce liver injury [5,6], raising concerns regarding its safety and limiting its medical applications.

Hepatic toxicity caused by EGCG supplements has been documented in both preclinical studies and clinical reports [7,8], highlighting the need for strategies to prevent EGCG-induced hepatotoxicity. However, no cases of liver injury resulting from tea consumption, even with high doses, could be found [9], suggesting that some ingredients in tea might alleviate the side effects of EGCG. A promising compound is L-theanine, a unique non-protein amino acid primarily found in tea leaves. L-theanine is an amino acid analog of the proteinogenic amino acids L-glutamate and L-glutamine (Figure S1B). It makes up 1–2% of the dry weight of fresh tea leaves and contributes to the favorable umami taste of tea [10,11]. L-theanine also offers several physiological effects, including relaxation, cognitive enhancement, and potential neuroprotective properties [12]. Moreover, emerging evidence suggests that L-theanine could perform antioxidant, anti-inflammatory, and hepatoprotective activities [12,13,14], making it an intriguing candidate for counteracting EGCG-induced hepatic toxicity.

While several studies have investigated the individual effects of EGCG and L-theanine on liver function, few have explored their interactions in the context of hepatic toxicity. Despite the growing interest in the hepatoprotective potential of L-theanine, it remains unclear whether it can alleviate EGCG-induced liver injury. Understanding the interplay between EGCG and L-theanine may yield valuable insights into the mechanisms underlying EGCG-induced hepatotoxicity and contribute to the development of novel strategies for its prevention and management.

This study aims to confirm the protective effect of L-theanine against EGCG-induced acute hepatic toxicity in an in vivo model. Through a metabolomic approach, we hope to elucidate the potential synergistic or antagonistic interactions between L-theanine and EGCG, shed light on the mechanisms involved in their health effects, and evaluate the therapeutic potential of L-theanine. Our findings may provide important guidance for the safe and effective use of EGCG-containing products and contribute to the development of L-theanine as a dietary supplement or pharmacological intervention aimed at preserving liver health.

## 2. Materials and Methods

### 2.1. Chemicals

L-theanine (purity > 98%) and EGCG (purity > 95%) were generously provided by Hunan Sanfu Biological Technology Co., LTD (Changsha, China). L-theanine and EGCG were dissolved in distilled water (assisted by ultrasonic if needed) just before intragastric administration (i.g.) to mice.

### 2.2. Animal Experiments

All the experimental procedures and animal care in this study were designed strictly according to the animal care standards formulated by the Ethical Inspection Committee of Hunan Agricultural University in 2021 (registry number: 2138). Male ICR mice (4 weeks of age and weighing 18–22 g) were obtained from the Hunan Slack Jing-da Experimental Animal Co., Ltd. (Changsha, China). The mice were acclimatized for 1 week before the experiment was conducted. Animals were housed in a controlled environment (20–25 °C, 50–70% humidity) with a 12 h light/dark cycle and had free access to regular feed and drinking water. Feed, water, and bedding were changed every two days to keep the cages clean and hygienic.

Forty mice were randomly divided into four groups (*n* = 10 for each group): control group (Con), EGCG group (EGCG), L-theanine plus EGCG (LT + EGCG), and L-theanine group (LT). Mice in the Con and EGCG groups received sterile distilled water daily, while mice in LT + EGCG and LT groups were administrated 300 mg/kg L-theanine daily via i.g. for 7 days. The intragastric administration was applied by a trained researcher (L.Y.) at 9 a.m. every day. On the 7th day, 30 min after the last administration, mice in the EGCG and LT + EGCG groups received 1000 mg/kg EGCG via i.g., while the other two groups received distilled water. The dose of EGCG was decided according to the published study [6] and our pre-experiment, which demonstrated that 1000 mg/kg EGCG might be the lethal dose of 50% for ICR mice (Figure S2A). Between 200 and 400 mg/kg of L-theanine is the functional dose widely used in mice models [15,16,17], which was also confirmed in our pre-experiment (Figure S2B). Thus, 300 mg/kg L-theanine is considered a reasonable dose for daily use and was selected for this study.

Twenty-four h after EGCG administration, the mice were anesthetized with sodium pentobarbital and sacrificed. Blood samples were collected from the eye socket. The serum fraction was separated via centrifugation (3500 rpm/min, 10 min) and was stored at −80 °C until analysis. Liver tissues were collected as soon as possible and washed with 4 °C saline, then frozen in liquid nitrogen and stored at −80 °C until further use.

### 2.3. Histopathological Examination

Immediately after dissection, liver slices from individual mice were fixed in 10% buffered formaldehyde, embedded in paraffin, cut into 4 μm thick sections, stained with hematoxylin and eosin (HE), photographed under microscopy, and finally underwent a histopathological examination. Liver injury was decided by an experienced researcher (H.Z.), who was blinded to the samples.

### 2.4. Biochemical Assays

Activities of alanine aminotransferase (ALT) and aspartate aminotransferase (AST) in the serum samples were measured through spectrophotometric methods according to the instructions provided with the commercial assay kits (Nanjing Jiancheng Bioengineering Institute, Nanjing, China). Liver samples were prepared into homogenates in saline and used for the quantification of the levels of superoxide dismutase (SOD), antioxidant enzymes catalase (CAT), glutathione peroxidase (GSH-Px), glutathione (GSH), and malondialdehyde (MDA), through commercially available kits (Nanjing Jiancheng Bioengineering Institute, Nanjing, China).

### 2.5. Enzyme-Linked Immunosorbent Assay

4-hydroxynonenal (4-HNE), tumor necrosis factor-a (TNF-α), and the inflammatory cytokines interleukin-6 (IL-6) in the serum of mice were determined by commercial ELISA kits (Cusabio Life Science, Wuhan, China).

### 2.6. RNA Preparation and Quantification

The total RNA was isolated from each liver sample through the SteadyPure Universal RNA Extraction Kit (Accurate Biotechnology Co., Ltd., Changsha, China), according to the manufacturer’s protocol. The integrity of total RNA was examined by 1% agarose gel electrophoresis. RNA concentration was evaluated by Nanodrop 2000 (Thermo Fisher Scientific Inc., Waltham, MA, USA). cDNA was prepared by reverse transcription of RNA by using Evo M-MLV RT Premix for quantitative polymerase chain reaction (qPCR) (Accurate Biotechnology Co., Ltd., Changsha, China). qPCR was performed on QuantStudio 3 Flex system (Thermo Fisher Scientific Inc., Waltham, MA, USA) using SYBR Green Premix Pro Taq HS qPCR Kit (Accurate Biotechnology Co., Ltd., Changsha, China). The following cycling conditions were used: 95 °C for 30 s, followed by 40 cycles of 95 °C for 5 s and 60 °C for 30 s for amplification and quantification, respectively. The threshold cycle value (CT) of each qPCR reaction was determined. The relative expression value was calculated using actin beta (ACTB) as a reference gene. Fold changes were normalized using the 2^−ΔΔCt^ method. The primer sequences for the targeted mouse genes, including ACTB, nuclear factor erythroid-related factor 2 (Nrf2), heme oxygenase-1 (HO-1), NAD(P)H quinone dehydrogenase 1 (NQO1), glutamate-cysteine ligase catalytic subunit (GCLC), glutamate-cysteine ligase modifier subunit (GCLM), glutathione S-transferase mu 1 (GSTm1), and glutathione S-transferase mu 2 (GSTm2) were synthesized by Sangon Biotech (Shanghai, China) and shown in Table S1.

### 2.7. Metabolomic Analysis

Metabolite extraction was primarily performed according to previously reported methods [18]. Briefly, 25 mg liver tissues were diluted with 800 µL of precooled extraction reagent (methanol: acetonitrile: water, 2:2:1). After homogenizing for 5 min, samples were sonicated for 10 min and incubated at −20 °C for 1 h. Then, the samples were centrifuged for 15 min (25,000 rpm, 4 °C), and the supernatant was then collected and underwent vacuum freeze drying. The metabolites were then redissolved in 600 µL of 70% acetonitrile and sonicated for 10 min at 4 °C. After centrifuging for 15 min at 25,000 rpm, the supernatants were transferred to autosampler vials for LC-MS analysis.

Waters 2D UPLC (Waters, Milford, MA, USA) tandem Q Exactive high-resolution mass spectrometer (Thermo Fisher Scientific, Waltham, MA, USA) was used for the separation and detection of metabolites. A Waters ACQUITY UPLC BEH Amide column (1.7 μm, 2.1 mm × 100 mm, Waters, USA) was used for the chromatography separation at 30 °C. The mobile phase consisted of 0.1% (*v*/*v*) formic acid and 10 mM ammonium formate, and the mobile phases A and B contained 95% acetonitrile and 50% acetonitrile, respectively. The gradient conditions were set as follows: 0–0.5 min, 2% B; 0.5–12 min, 2–50% B; 12–14 min, 98% B; 14–16 min, 98% B; 16–16.1 min, 98–2% B; and 16.1–18 min, 2% B. The injection volume was 2.0 μL, and the flow rate was set to 0.35 mL/min.

The mass spectrometric settings for positive/negative ionization modes were as follows: the spray voltage was set to 3.8 kV in positive mode and 3.2 kV in negative mode; the sheath gas flow rate was 40 arbitrary units (arb); the aux gas flow rate was 10 arb; the aux gas heater temperature was 350 °C; and the capillary temperature was 320 °C. Metabolites were detected by full scan mass analysis from *m*/*z* 70–1050. The stepped normalized collision energy was set to 15/30/45.

### 2.8. Data Processing and Statistical Analysis

The raw data collected from LC-MS/MS were imported into the Compound Discoverer 3.1 (Thermo Fisher Scientific, Waltham, MA, USA) for data processing. The main steps included peak extraction, peak alignment, and compound identification. The identification and annotation of metabolites was a combination of the search results from the mzCloud database (https://www.mzcloud.org/, accessed on 25 August 2021), the human metabolome database (HMDB) (https://hmdb.ca/metabolites, accessed on 25 August 2021), and the Kyoto Encyclopedia of Genes and Genomes (KEGG) database (http://www.genome.jp/kegg/, accessed on 25 August 2021). The main parameters for metabolite identification were as follows: precursor mass tolerance < 5 ppm, fragment mass tolerance < 10 ppm, RT tolerance < 0.2 min.

The preprocessed data were imported and analyzed using MetaboAnalyst tools (https://www.metaboanalyst.ca/, accessed on 21 March 2024). Briefly, features were filtered based on QC samples with RSDs greater than 20%, and with interquantile range > 40%. The filtered data were normalized by sum and transformed to log (base 10) form. The normalized data were further analyzed through *t*-tests and Orthogonal Partial Least Squares–Discriminant Analysis (OPLS-DA) to identify the differential expressed metabolites. Compounds with VIP value > 1, fold change > 2 or <0.5, and *p*-value < 0.05 were defined as significantly different. Pathway analysis of metabolites underwent the hypergeometric test, with topology analysis using the “Out-degree Centrality” method.

Other statistical data were expressed as means ± standard error of the mean (SEM). All the comparisons to determine differences were performed by applying a two-way analysis of variance (ANOVA), followed by Tukey’s test. Statistical significance was set as follows: * *p* < 0.05, ** *p* < 0.01, and *** *p* < 0.001.

## 3. Results

### 3.1. L-Theanine Reduced EGCG-Induced Liver Injury

**Figure 1:**
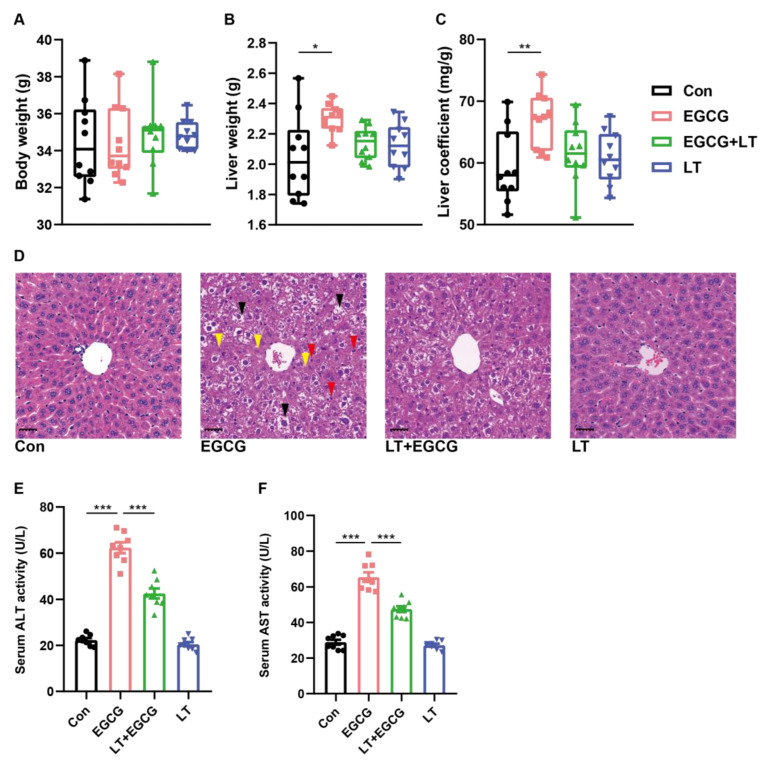
L-theanine reduced EGCG-induced liver injury. Body weight (**A**), liver weight (**B**), and liver coefficient (**C**) of mice (*n* = 10). (**D**) Representative photograph of HE-stained liver tissues (scale bar = 50 μm), arrows in black: cytoplasm vacuolization and edema, arrows in red: neutrophil infiltration, arrows in yellow: necrosis. Activity of serum ALT (**E**) and AST (**F**); data were presented as mean ± SEM (*n* = 8). * *p* < 0.05, ** *p* < 0.01, and *** *p* < 0.001.

A single dose of 1000 mg/kg EGCG significantly increased the liver weight and the liver coefficient of mice, with no obvious effects on body weight (Figure 1A–C), indicating that edema or hemorrhage might occur. The HE-stained liver section of the EGCG group showed considerable inflammatory alterations characterized by neutrophil infiltration, cytoplasm vacuolization, edema, and necrosis (Figure 1D). Additionally, the significantly higher activities of ALT and AST in the serum of the EGCG group also confirmed the liver injury caused by high-dose EGCG (Figure 1E,F). Pretreatment with L-theanine reduced liver injury to a certain extent and significantly alleviated EGCG-induced transaminitis, while no obvious effects could be seen resulting from L-theanine alone (Figure 1B–F).

### 3.2. L-Theanine Alleviated EGCG-Induced Oxidative Stress

**Figure 2:**
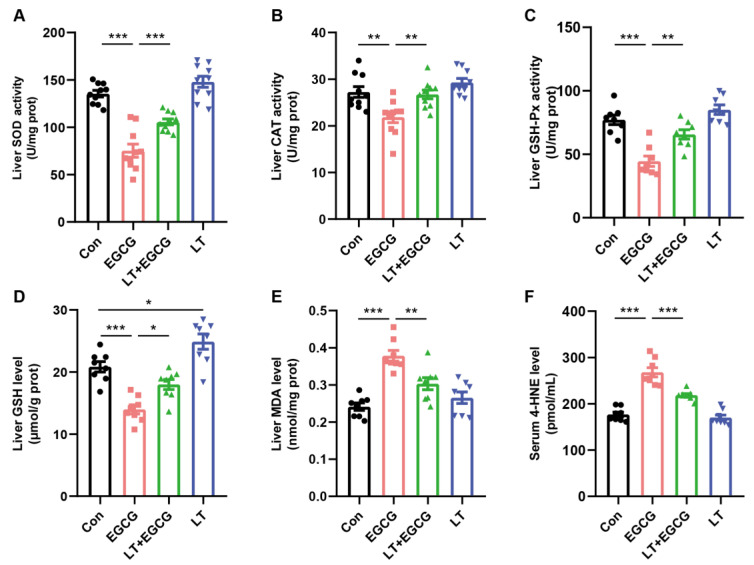
L-theanine alleviated EGCG-induced oxidative stress. Hepatic activity of SOD (**A**), CAT (**B**), and GSH-Px (**C**); GSH (**D**) and MDA (**E**) contents of liver tissues; (**F**) Serum levels of 4-HNE. Data were presented as mean ± SEM (*n* = 8). * *p* < 0.05, ** *p* < 0.01, and *** *p* < 0.001.

To decide whether the liver injury induced by EGCG resulted from increased oxidative stress, various oxidative stress indexes were quantified. The activity of SOD, CAT, and GSH-Px, three common antioxidative enzymes, were significantly inhibited by EGCG (Figure 2A–C). Moreover, the content of GSH in the liver was significantly decreased (Figure 2D). By contrast, the decreased activity and amount of enzymatic and non-enzymatic antioxidants could be effectively prevented by pretreatment of L-theanine (Figure 2A–D).

As a result of oxidative damage, lipid peroxidation products such as MDA and 4-HNE accumulated in the liver. Compared to the control group, MDA and 4-HNE levels significantly increased after EGCG administration (Figure 2E,F). Pretreatment of L-theanine significantly inhibited the over-production of these lipid peroxidation products, suggesting that L-theanine could effectively reduce the level of oxidative stress induced by high-dose EGCG.

Continuous administration of 300 mg/kg L-theanine did not result in the fluctuation of oxidative status (Figure 2A–F). However, we noticed that the GSH content was significantly increased in the liver tissue of mice in the LT group (Figure 2D), which may contribute to the preventive effects of L-theanine on EGCG-induced oxidative stress.

### 3.3. L-Theanine Alleviated EGCG-Induced Inhibition of Nrf2-Driven Downstream Signals

**Figure 3:**
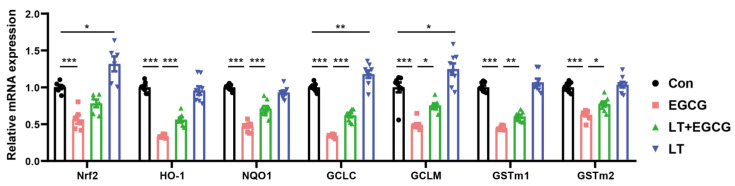
L-theanine alleviated EGCG-induced inhibition of Nrf2-driven downstream signals. Data are presented as mean ± SEM (*n* = 8, except for Nrf2 *n* = 6). * *p* < 0.05, ** *p* < 0.01, and *** *p* < 0.001.

As an important transcription factor regulating oxidative stress, Nrf2 is a major hub for initiating endogenous antioxidant responses. Activation of Nrf2 can promote the expression of GSH synthesis-related genes, including glutathione reductase (GR), GCL, and GST [19]. Since L-theanine administration resulted in notable improvement of GSH content (Figure 2D), the mRNA content of Nrf2 in the liver tissues of mice was measured. As a result, the transcription of Nrf2 was significantly reduced by EGCG, and pretreatment of L-theanine could alleviate the impact of EGCG. Further measurement of the mRNA content of Nrf2-driven downstream genes showed a similar expression trend. A single dose of 1000 mg/kg EGCG resulted in significant transcriptional downregulation of HO-1, NQO1, GCLC, GCLM, GSTm1, and GSTm2, compared to the control (Figure 3).

By contrast, pretreatment of L-theanine could alleviate EGCG-induced transcriptional downregulation of these genes (Figure 3). Moreover, significantly increased mRNA expression of GCLC and GCLM was found in the LT group, which may contribute to the increased amount of GSH shown in Figure 2D.

### 3.4. Metabolomic Revealed Metabolite Differences between Groups

**Figure 4:**
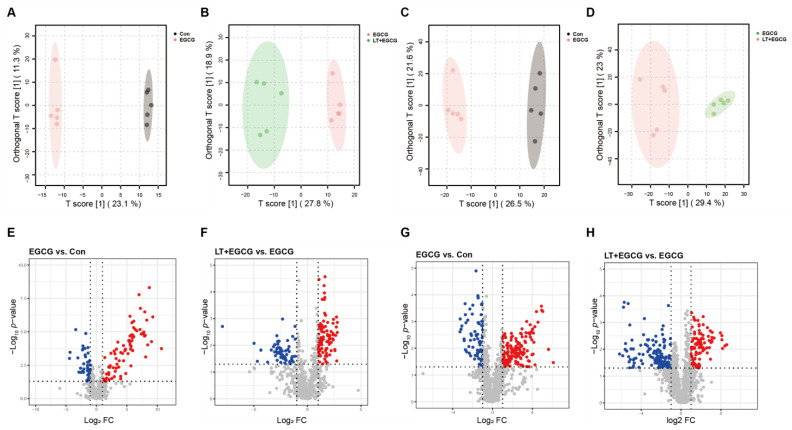
Identification of differentially regulated metabolites. Score plots based on OPLS-DA models for (**A**) EGCG group versus Con group, positive ion mode; (**B**) LT + EGCG group versus EGCG group, positive ion mode; (**C**) EGCG group versus Con group, negative ion mode; and (**D**) LT + EGCG group versus EGCG group, negative ion mode. Volcano plot showing the differentially regulated metabolites (red dots: significantly up-regulated; blue dots: significantly down-regulated; grey dots: insignificantly regulated) for (**E**) EGCG group versus Con group, positive ion mode; (**F**) LT + EGCG group versus EGCG group, positive ion mode; (**G**) EGCG group versus Con group, negative ion mode; and (**H**) LT + EGCG group versus EGCG group, negative ion mode.

To further analyze the effects of L-theanine on EGCG-induced liver injury at the metabolic level, a non-targeted metabolomic approach was applied to the liver samples in the Con, EGCG, and LT + EGCG groups. A total of 3673 compounds were identified (1567 from positive ion mode and 2106 from negative ion mode), among which 1393 compounds had information annotated (Tables S2 and S3). The samples in different groups were effectively separated in the OPLS-DA models for both positive and negative modes (Figure 4A–D), indicating significant metabolic changes were induced by EGCG and L-theanine administration. Compared to the control group, 233 compounds were significantly upregulated, and 113 compounds were significantly downregulated in the EGCG group. While compared to the EGCG group, 196 compounds were significantly upregulated, and 207 compounds were significantly downregulated in the LT + EGCG group (Figure 4E–H, Tables S4 and S5).

**Figure 5:**
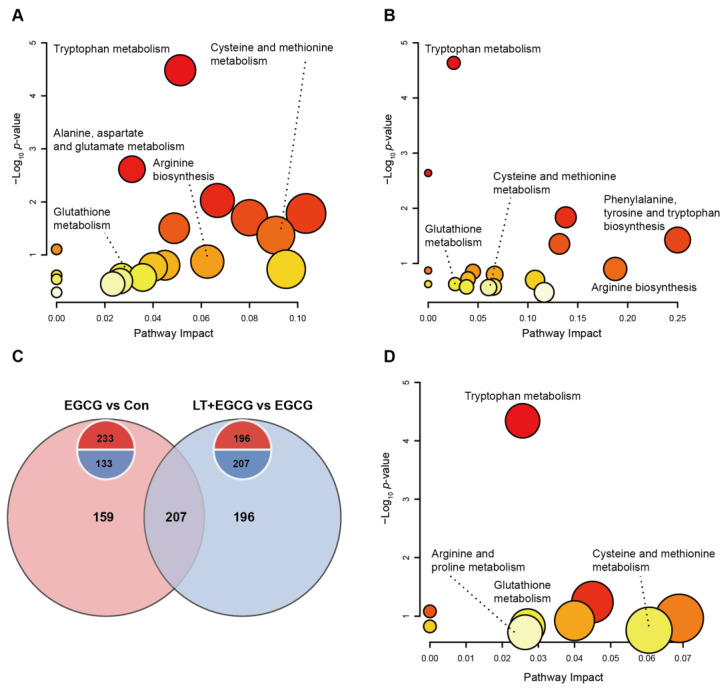
Pathway analysis of differentially regulated metabolites. (**A**) Pathway affected by the differentially regulated metabolites between EGCG group and Con group. (**B**) Pathway affected by the differentially regulated metabolites between LT + EGCG group and EGCG group. (**C**) Venn diagram for the intersection of differentially regulated metabolites from two comparisons. (**D**) Pathway affected by the intersection of differentially regulated metabolites. The color of each circle is proportional to the *p*-value, *p*-value decreases from light to dark. The size of each circle is proportional to the pathway impact value.

Through pathway analysis of the significantly regulated metabolites, we found that besides GSH metabolism, many metabolic pathways for amino acids (such as tryptophan, cysteine, methionine, arginine, alanine, aspartate, and glutamate) were affected by high-dose EGCG administration (Figure 5A). Pretreatment of L-theanine could alleviate the adverse effects induced by EGCG on these pathways (Figure 5B). There were 207 compounds in the intersection of EGCG-induced differential metabolites and L-theanine-induced differential metabolites between the LT + EGCG group and the EGCG group (Figure 5C). Two hundred and four of the overlapped metabolites showed inverse trends, indicating that L-theanine could protect liver tissue from EGCG-induced fluctuation of homeostasis (Table S6). Similarly, while the metabolism of arginine, proline, cysteine, methionine, and GSH was enriched, the most significantly affected pathway by these overlapped compounds was the tryptophan metabolism (Figure 5D).

### 3.5. L-Theanine Protected the Liver from EGCG-Induced Inflammatory Response

**Figure 6:**
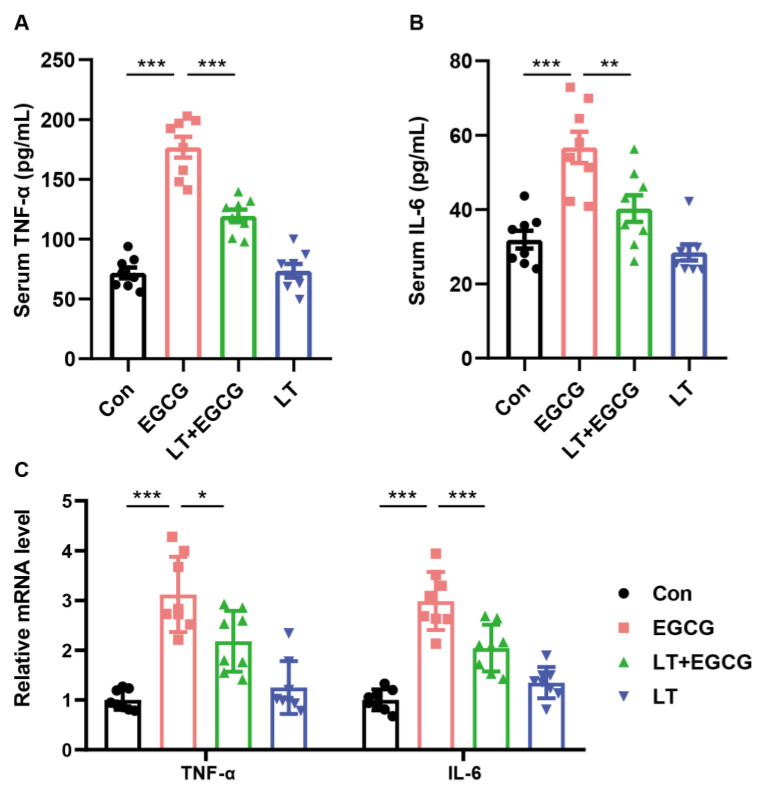
L-theanine reduced the EGCG-induced inflammatory response. Serum levels of TNF-α (**A**) and IL-6 (**B**). (**C**) Relative hepatic mRNA levels of TNF-α and IL-6. Data are presented as mean ± SEM (*n* = 8). * *p* < 0.05, ** *p* < 0.01, and *** *p* < 0.001.

Tryptophan is an essential amino acid and affects various pathophysiological processes, including inflammatory responses and oxidative stress [20]. Thus, two primary inflammatory cytokines, TNF-α and IL-6, were measured to study the difference in inflammatory response among groups and verify the metabolomic results. As a result, the contents of TNF-α and IL-6 in serum significantly increased by 1000 mg/kg EGCG (Figure 6A,B). Moreover, the relative mRNA levels of TNF-α and IL-6 in liver tissues showed consistent expression tendency (Figure 6C). Pretreatment of L-theanine significantly mitigated EGCG-induced inflammatory response (Figure 6A–C), indicating that L-theanine could reduce inflammation through the regulation of tryptophan metabolism and inflammatory factors.

## 4. Discussion

The present study aimed to investigate the potential protective effects of L-theanine against EGCG-induced hepatic toxicity. Our findings could contribute to the studies on the health effects of green tea constituents and provide insights into the interplay between EGCG and L-theanine in modulating liver function.

High doses of orally administered EGCG can induce liver damage and promote inflammatory responses, which are well documented in many studies, presenting with marked hepatotoxicity in the form of acute hepatitis [6,21,22]. Consistent with the existing evidence, 1000 mg/kg EGCG resulted in obvious liver damage in ICR mice. Moreover, our results demonstrated that L-theanine supplementation attenuated EGCG-induced hepatotoxicity in vivo. Specifically, we observed a significant reduction in markers of liver injury, including serum levels of important hepatic enzymes such as ALT and AST, in mice pretreated with L-theanine compared to those treated with EGCG alone. These findings suggest that L-theanine could exert hepatoprotective effects by mitigating the hepatic damage induced by high-dose EGCG.

The specific mechanisms underlying the protective effect of L-theanine against EGCG-induced hepatic toxicity are not fully understood but probably involve the antioxidant pathways, as increased GSH contents have resulted from L-theanine administration. GSH-mediated biotransformation in the liver is the most common way to eliminate small xenobiotics [23]. GSH could scavenge free radicals directly or act as a substrate by antioxidant enzymes such as GST [24], which catalyzes the conjugation of GSH with xenobiotic compounds and plays a vital role in protecting cells against oxidative stress. Nrf2 is a primary transcription factor that acts as an important hub of endogenous antioxidant defense systems, including GSH recycling and synthesis [25,26,27]. When cells are under oxidative stress, Nrf2 is released in the cytoplasm and translocated into the nucleus to enable a series of antioxidant/phase II detoxifying enzymes expression [26,28]. In this study, the inhibition of Nrf2 and downstream targets (HO-1, NQO-1, GCLC, GCLM, and GST) by EGCG was significantly reduced by L-theanine pretreatment, indicating that L-theanine had a protective effect on EGCG induced oxidative damage through Nrf2 related pathways. A small number of studies have shown that L-theanine could promote Nrf2 expression in pathological conditions [29,30], which is consistent with our results in the high-dose EGCG in vivo model. Moreover, administration of L-theanine resulted in significantly increased expression of GCLC and GCLM, which are rate-limiting enzymes in endogenous synthesis of GSH [24] and contributed to the increased GSH content in the liver of mice in the LT group. All the evidence indicated that GSH metabolism played a critical role in the interaction between EGCG and L-theanine.

Through pathway analysis of the metabolomic data, we found that besides GSH metabolism, many metabolic pathways for amino acids (such as tryptophan, cysteine, methionine, arginine, etc.) were affected by high-dose EGCG. Particularly, we pointed out for the first time that tryptophan metabolism may play an important role in high-dose EGCG-induced liver injury. Tryptophan metabolites can have pro-inflammatory or anti-inflammatory effects, and their imbalance contributes to the pathogenesis of liver injury and inflammation [20,31]. Moreover, imbalances in tryptophan metabolism have been linked to various liver diseases, including non-alcoholic fatty liver disease, alcoholic liver disease, and liver cirrhosis [32,33]. Tryptophan supplementation could reduce neuroinflammation by downregulating the levels of TNF-α, IL-6, IL-1b, and TLR-4 in mouse models of chronic, unpredictable mild stress [20,34]. Moreover, tryptophan could relieve liver damage, increase the antioxidant capacity, and reduce inflammation in piglets [35]. In the present study, the serum content of TNF-α and IL-6 was increased by EGCG, as well as the mRNA expression in the liver tissue, indicating increased inflammatory reactions. By contrast, pretreatment of L-theanine reduced the EGCG-induced increase of TNF-α and IL-6. Consistent with previous studies, these results indicated that L-theanine could protect EGCG-induced inflammation response, probably through regulation of tryptophan metabolism.

However, the limitations of this study should be noted. The doses of EGCG and L-theanine used in this study may not reflect real-world consumption patterns, and the use of animal models may not fully recapitulate the complexity of human liver physiology and metabolism. The proportion of EGCG and L-theanine in a green tea infusion may vary depending on many factors, such as tea leave quality, type, and brewing time. However, an 8-ounce cup of tea usually contains 50–100 mg EGCG and 5–20 mg L-theanine [11,36,37]. Thus, it is impossible to cause liver injury by simply tea consumption, whereas EGCG supplements do cause liver burden and liver injury in some special cases, such as dietary restriction during weight control [38,39]. Additionally, although we pointed out that the combination use of L-theanine and EGCG could alleviate the potential side effects of EGCG, and the modulation of tryptophan metabolism could play an important role during the procedure, further studies are still needed to be carried out to reveal the specific mechanism.

## 5. Conclusions

This study demonstrated that L-theanine supplementation could attenuate EGCG-induced hepatic toxicity in ICR mice by reducing oxidative stress and inflammation response through activation of Nrf2-mediated endogenous antioxidant system and modulation of tryptophan metabolism. These results could provide valuable insights into the potential therapeutic applications of L-theanine and highlight the importance of the interactions between dietary components. Further research should focus on elucidating the specific mechanism underlying the protective effects of theanine and evaluating its efficacy in clinical trials.

## Data Availability

Data is contained within the article or Supplementary Material.

## Supplementary Materials

The following supporting information can be downloaded at: https://www.mdpi.com/article/10.3390/foods13071121/s1. Figure S1: Chemical structure of EGCG and L-theanine; Figure S2: Survivorship curve of mice under different dosages of EGCG and pretreatment of L-theanine; Table S1: Primer sequences used for qPCR; Table S2: Compounds identified in the positive ion mode; Table S3: Compounds identified in the negative ion mode; Table S4: Significantly expressed metabolites in EGCG versus Con group; Table S5: Significantly expressed metabolites in LT + EGCG versus EGCG group; Table S6: Intersection of two comparisons.
