# Supplementary material for: The Protective Effects of L-Theanine against Epigallocatechin Gallate-Induced Acute Liver Injury in Mice
Source: Foods. 2024 Apr 7;13(7):1121. doi: 10.3390/foods13071121 (PMC11011850; doi:10.3390/foods13071121)
Supplement: Supplementary file 1 [file foods-13-01121-s001.zip › Figure S2.pdf]

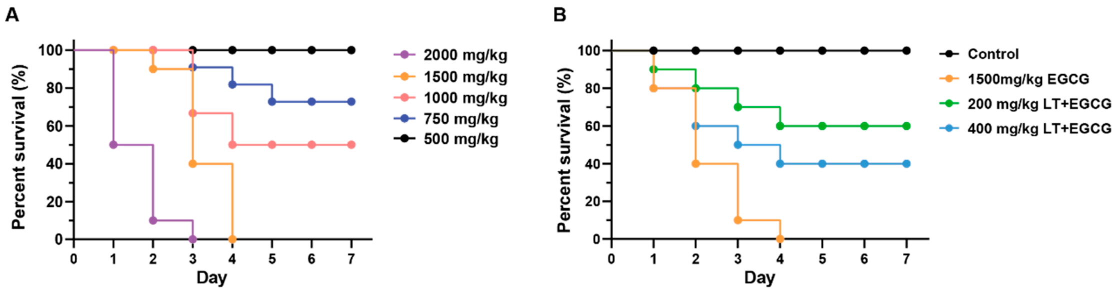

Figure S2. Survivorship curve of mice under different dosages of EGCG (A) and pretreatment of L-theanine (B)
