# Supplementary figures and images for: The Protective Effects of L-Theanine against Epigallocatechin Gallate-Induced Acute Liver Injury in Mice
Source: Foods. 2024 Apr 7;13(7):1121. doi: 10.3390/foods13071121 (PMC11011850; doi:10.3390/foods13071121)

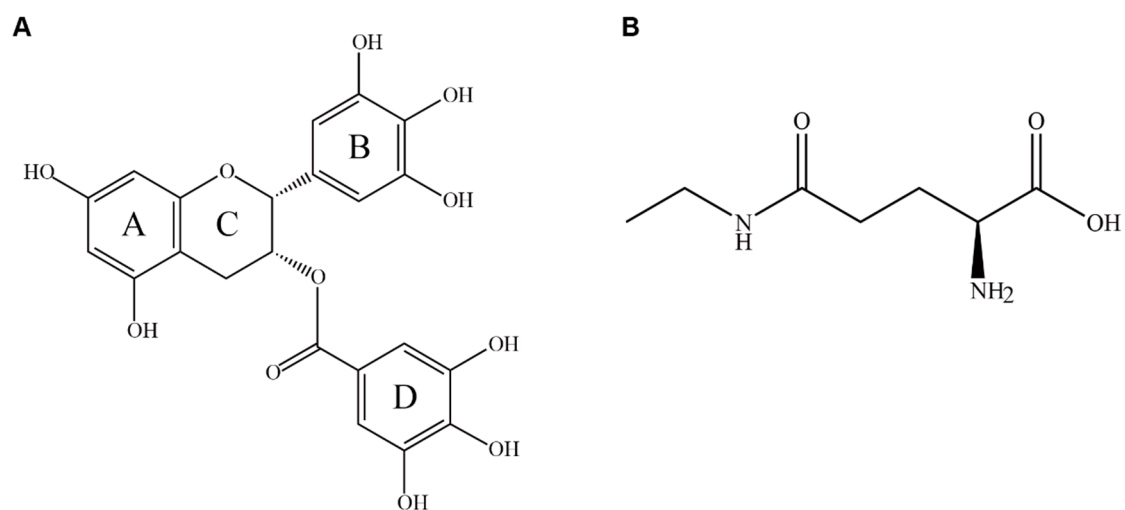

**Figure S1.** Chemical structure of epigallocatechin-3-gallate (A) and L-theanine (B)

Supplement: Supplementary file 1 [file foods-13-01121-s001.zip › Figure S1.pdf]
